# What the SIF Is Happening—The Role of Intracellular *Salmonella*-Induced Filaments

**DOI:** 10.3389/fcimb.2017.00335

**Published:** 2017-07-25

**Authors:** Katelyn Knuff, B. Brett Finlay

**Affiliations:** ^1^Michael Smith Laboratories, University of British Columbia Vancouver, BC, Canada; ^2^Department of Microbiology and Immunology, University of British Columbia Vancouver, BC, Canada; ^3^Department of Biochemistry and Molecular Biology, University of British Columbia Vancouver, BC, Canada

**Keywords:** *Salmonella* typhimurium, *Salmonella*-induced filaments, *Salmonella*-containing vacuole, multiple contact sites, endosomal system

## Abstract

A common strategy among intracellular bacterial pathogens is to enter into a vacuolar environment upon host cell invasion. One such pathogen, *Salmonella enterica*, resides within the *Salmonella*-containing vacuole (SCV) inside epithelial cells and macrophages. *Salmonella* hijacks the host endosomal system to establish this unique intracellular replicative niche, forming a highly complex and dynamic network of *Salmonella*-induced filaments (SIFs). SIFs radiate outwards from the SCV upon onset of bacterial replication. SIF biogenesis is dependent on the activity of bacterial effector proteins secreted by the *Salmonella*-pathogenicity island-2 (SPI-2) encoded type III secretion system. While the presence of SIFs has been known for almost 25 years, their precise role during infection remains elusive. This review summarizes our current knowledge of SCV maturation and SIF biogenesis, and recent advances in our understanding of the role of SIFs inside cells.

## Introduction

*Salmonella enterica* serovars are Gram-negative bacterial pathogens capable of causing enteric disease in all vertebrates. *S. enterica* serovars are associated with illnesses ranging from gastroenteritis to typhoid fever caused by non-typhoidal *Salmonella* (NTS) serovars and *S. enterica* subsp. *enterica* serovar Typhi, respectively (Haraga et al., 2008; Keestra-Gounder et al., 2015). Salmonellae are facultative intracellular pathogens that reside within a unique membrane-bound compartment termed the *Salmonella*-containing vacuole (SCV) following host cell invasion. Entry into a bacteria-containing vacuole (BCV) is seen in diverse intracellular pathogens including *Legionella pneumophila, Shigella flexneri, Francisella tularensis, Mycobacterium tuberculosis*, and *Edwardsiella* spp (Silva, 2012). Like *Salmonella, Legionella* and *M. tuberculosis* largely remain and replicate within a BCV, while *Shigella, Francisella*, and *Edwardsiella* escape their vacuoles and replicate within the host cell's cytoplasm (Okuda et al., 2006; Santos and Enninga, 2016). This review focuses on intravacuolar *Salmonella*, but a small portion of *Salmonella* (around 10%) escape the SCV and enter a hyper-replicative state within the cytosol of epithelial cells (Knodler et al., 2010; Malik-Kale et al., 2012; Yu et al., 2014; Santos et al., 2015).

*S. enterica* forms networks of *Salmonella*-induced tubules (SITs) radiating outwards from the SCV, throughout the host cell's cytoplasm. *Salmonella*-induced filaments (SIFs) are the most abundant, and best studied, form of SIT. Intravacuolar replication accompanies formation of SIFs which are endosomal-tubule extensions characterized by the presence of lysosomal glycoproteins and other endocytic markers (Garcia-del Portillo et al., 1993; Beuzón et al., 2000). Other SIT types include *Salmonella*-induced secretory carrier membrane protein 3 (SCAMP3) tubules (SISTs) (Mota et al., 2009), LAMP1-negative tubules (Schroeder et al., 2010), sorting nexin 3 tubules, and spacious vacuole-associated tubules (Schroeder et al., 2011).

To create and maintain the SCV, *Salmonella* Typhimurium, the model organism for NTS infections, uses specialized secretion systems to inject at least 43 bacterial protein “effectors” into the host cell cytoplasm (Galán, 2001; Figueira and Holden, 2012; LaRock et al., 2015). Effectors are secreted by two distinct type III secretion systems (T3SSs) encoded on *Salmonella* pathogenicity islands 1 and 2 (SPI1-T3SS and SPI2-T3SS, respectively). While SPI1-T3SS-secreted effectors enable host cell invasion and SCV biogenesis, the SPI2-T3SS-secreted effectors are associated with SCV maturation, SIF biogenesis, and promoting survival and replication (McGhie et al., 2009). While SIFs have been extensively studied, only recently have we begun to understand their intracellular role. SIF studies have primarily been conducted in HeLa cells, and a small number in the murine macrophage cell line RAW264.7. The role of SIFs is unclear in macrophages as they have yet to be demonstrated in primary macrophage cells. This review summarizes our current knowledge, and recent advances in our understanding, of SCV maturation and SIF biogenesis in epithelial cells.

## SCV maturation: *Salmonella* makes a home for itself

The early stages of SCV formation and maturation (“early SCVs”) in HeLa cells resemble early endosomes with markers for endocytic sorting and recycling pathways and subsequent maturation pathways, partially owing to the activities of T3SS-secreted effectors. The SPI1-T3SS-secreted effector SopB maintains high levels of SCV membrane-associated phosphatidyl-3-phosphate during initial stages of SCV maturation (Hernandez et al., 2004). SopB recruits the small GTPase Rab5, which in turn, recruits early endosome antigen 1 (EEA1) to the SCV membrane (Haraga et al., 2008; Mallo et al., 2008). Rab5 promotes docking and fusion of early endosomes to various targets, and regulates the conversion of early to late endosomes (Christoforidis et al., 1999; Huotari and Helenius, 2011). Early SCVs are also characterized by the endocytic markers transferrin receptor (TfnR), Rab4, and Rab11 (Steele-Mortimer et al., 1999; Smith et al., 2005). Rab4 regulates early sorting events in endosomes while Rab11 recycles membrane components between the plasma membrane and the Golgi (Sheff et al., 1999; Sönnichsen et al., 2000).

**Figure 1:**
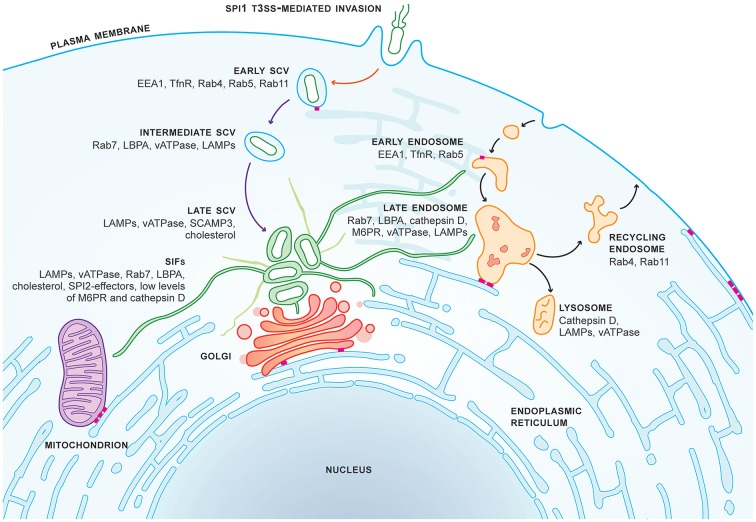
SCV maturation and SIF biogenesis in epithelial cells. *Salmonella* Typhimurium invades epithelial cells in a SPI1-T3SS-dependent manner and specifically resides in the *Salmonella*-containing vacuole (SCV) within the host cell. Studies primarily in HeLa cells have revealed that formation of the early SCV is dependent on SPI1-T3SS-secreted effectors (red arrow) and occurs within 15 min post-invasion (p.i.). SCV maturation is dependent on SPI2-T3SS-secreted effectors (purple arrows). The late SCV is formed by 3–4 h.p.i. SCV maturation closely resembles, but is distinct from, endosome maturation (black arrows). The SCV is located next to the Golgi by 8 h.p.i., coinciding with the formation of *Salmonella*-induced filaments (SIFs, green tubules). SIFs form an extensive network throughout the host cell facilitating interactions with host organelles. The tubular endoplasmic reticulum network (blue tubules) forms multiple contact sites (MCSs, pink bars) with organelles, the plasma membrane, and the early SCV.

SCV maturation, like endosome maturation, is marked by the rapid loss of early-, sorting-, and recycling-membrane markers, and acquisition of the late endosomal markers Rab7, lysosomal associated membrane proteins (LAMPs) 1, 2, and 3, and vATPase (see Figure 1 for comparison of SCV and endosome maturation) (Garcia-del Portillo and Finlay, 1995; Méresse et al., 1999; Steele-Mortimer et al., 1999; Beuzón et al., 2000; Drecktrah et al., 2007). SCVs acquire the late endosome markers Rab7 and Rab9, but are not enriched for the characteristic late endosomal/lysosomal markers cathepsin D, lysobisphosphatidic acid (LBPA), and mannose-6-phosphate receptor (M6PR) (Méresse et al., 1999; Brumell et al., 2001b; Knodler and Steele-Mortimer, 2005). This altered maturation program results from the activities of several SPI2-T3SS-secreted effectors and delayed interactions with late endocytic compartments.

The activities of the SPI2-T3SS-secreted effectors change the early SCV into a unique compartment permissive for bacterial replication, termed the “late SCV.” The SPI2-T3SS-secreted effectors SifA, SopD2, and SseJ are partially responsible for the SCV's unique maturation program. SifA complexes with the host factor SifA-and-Kinesin-Interacting-Protein (SKIP, also known as PLEKHM2); the SifA-SKIP complex binds and sequesters Rab9, inhibiting Rab9-dependent M6PR recruitment to the SCV membrane. Decreased M6PR recruitment to the SCV membrane decreases recruitment of lysosomal enzymes to the SCV, thereby protecting intracellular *Salmonella* from host defenses (McGourty et al., 2012). SopD2 further alters SCV maturation by directly impairing Rab7-dependent recruitment of the host trafficking-related effectors RILP (RAB-interacting-lysosomal protein) and FYCO1 (FYVE and coiled-coil domain containing protein 1). In uninfected cells, FYCO1 and RILP mediate plus- and minus- end-directed movement of vesicular cargo along microtubules, respectively (Jordens et al., 2001; Harrison et al., 2004; Pankiv et al., 2010). Inhibition of RILP- and FYCO1-mediated microtubule-based trafficking by SopD2 in infected cells thereby prevents delivery of the SCV to lysosomes (D'Costa et al., 2015). SseJ has two activities: phospholipase A activity, and glycerophospholipid:cholesterol acyltransferase activity (Lossi et al., 2008). Given these two enzymatic activities, SseJ may alter SCV lipid composition, thus altering the localization of lipid-bound proteins to the SCV (Sprong et al., 2001; Ruiz-Albert et al., 2002), and consequently mediating interactions with the host's endocytic pathway. *S*. Typhimurium is therefore able to alter the normal endosome maturation program to transform the late SCV into a unique niche within the host cell.

## SIF biogenesis

The process of SCV maturation from early- to late- SCV takes ~5 h post-invasion (h.p.i.) of HeLa cells. *Salmonella* replication coincides with full maturation of the late SCV and extension of SIFs. The remarkably dynamic process of SIF biogenesis results in a highly complex stabilized network of SIFs by 8 h.p.i., during which individual SIFs undergo extension, contraction, branching, and fusion with other SIFs (Drecktrah et al., 2008; Rajashekar et al., 2008). SIFs are the only type of SIT known to be marked by LAMPs (Garcia-del Portillo et al., 1993; Schroeder et al., 2011).

The same SPI2-T3SS-secreted effectors associated with SCV maturation are also associated with SIF biogenesis. These effectors are SifA, SseJ, SopD2, PipB2, SseF, SseG, SpvB, and SteA (Table 1). All eight of these effectors collectively contribute to at least one or more of the following roles within the host cell: promoting SIF biogenesis, perinuclear positioning of the SCV, maintaining stability and/or modifying the SCV membrane, and recruiting and/or regulating microtubule motor activity at the SCV membrane required for extension of SIFs along microtubules (Figueira and Holden, 2012; van der Heijden and Finlay, 2012; LaRock et al., 2015).

**Table 1:** SPI2-T3SS secreted effectors associated with SCV maturation and SIF biogenesis.

**Effector**	**Biochemical activity**	**Known host target(s)**	**Host subcellular localization/Effects**	**References**
SifA	Unknown	Rab7, Rab9, SKIP, RhoA, PLEKHM1	Localized to SIFs and SCV membranes, promotes SIF biogenesis, maintains SCV membrane stability, promotes kinesin-1-dependent movements along microtubules, enables continuous fusion of host vesicles to SCV membrane	Brumell et al., 2002; Harrison et al., 2004; Boucrot et al., 2005; Ohlson et al., 2008; Diacovich et al., 2009; Dumont et al., 2010; McGourty et al., 2012; Zhao et al., 2015
SseJ	Deacylase; transferase	RhoA, phospholipids, cholesterol	Localized to SCV membrane and SIFs, regulates SCV membrane dynamics, inhibits SIF biogenesis, esterifies cholesterol on SCV membrane	Ruiz-Albert et al., 2002; Freeman et al., 2003; LaRock et al., 2012; Kolodziejek and Miller, 2015
PipB2	Unknown	Kinesin-1	Localized to SIF and SCV membranes, recruits kinesin-1 to SCV membrane, reorganizes late endosome/lysosome compartments	Knodler and Steele-Mortimer, 2005; Henry et al., 2006
SopD2	GTPase-activating protein for Rab32	Rab7, Rab32	Localized to SCV membrane and host cell endosomes, inhibits host endocytic trafficking, antagonist of SifA in regulation of membrane dynamics and SIF biogenesis	Brumell et al., 2003; Schroeder et al., 2010; D'Costa et al., 2015; Spanò et al., 2016
SseF SseG	Unknown	Acyl-CoA binding domain containing 3	Integral membrane proteins localized to SIFs, tethers SCV to Golgi, converts single-membraned SIFs to double-membraned SIFs	Kuhle and Hensel, 2002; Kuhle et al., 2004; Deiwick et al., 2006; Krieger et al., 2014; Yu et al., 2016; Young et al., 2017
SteA	Unknown	Phosphatidylinositol 4-phosphate	Localized to membrane of SCV, SIFs, and SITs, control of SCV membrane dynamics	Van Engelenburg and Palmer, 2010; Domingues et al., 2014, 2016
SpvB	Actin ribosyltransferase	Unknown	Depolymerizes actin cytoskeleton, downregulates SIF biogenesis	Tezcan-Merdol et al., 2001; Birmingham et al., 2005

SifA classically has been considered the main driver of SIF biogenesis as Δ*sifA* mutants fail to induce SIFs in HeLa cells (Stein et al., 1996). Extensive vacuolation of LAMP1^+^ vesicles is observed in uninfected host cells transfected with SifA (Brumell et al., 2001a) suggesting that SifA alone is sufficient to induce endosomal tubulation resembling SIF-like structures. During infection, LAMP1 enrichment at the SCV membrane is enhanced by the C-terminal domain of SifA (Zhao et al., 2015). While SifA may be sufficient to induce endosomal tubulation, SseJ, when activated by GTP-bound RhoA, cooperates with SifA to promote formation of SIF-like structures (Ohlson et al., 2008; Christen et al., 2009). Observations that effector deletion mutants of any of the eight SPI2-T3SS effectors associated with SIF biogenesis have altered SIF morphology and/or frequency *in vitro* (Stein et al., 1996; Birmingham et al., 2005; Domingues et al., 2014; Rajashekar et al., 2014) suggests that all eight SIF-related effectors are required to produce wild-type SIFs within host cells.

Advances in fluorescence microscopy, transmission electron microscopy, and EM tomography have provided new insights into SIF biogenesis (Krieger et al., 2014). It was shown that nascent SIFs emerge as single-membrane tubules, dependent on SifA, and are thought to be of late endosomal or endolysosomal origin based on luminal content. Double-membrane SIFs were also observed in infected cells and are formed by the conversion of single- to double-membraned SIFs, a process dependent on both SseF and SseG. Double-membraned compartments are commonly observed during formation of autophagosomes (Rubinsztein et al., 2012). It is convenient to speculate that autophagy plays a role in SIF biogenesis since SIFs are double-membraned structures and multiple reports demonstrate autophagy controlling intracellular *Salmonella* (Hernandez et al., 2003; Birmingham and Brumell, 2006; Wild et al., 2011; Cemma and Brumell, 2012; Fujita et al., 2013). However, Krieger et al. (2014) found that autophagic machinery does not play a role in SIF biogenesis; instead, SIFs likely originate from another, SPI2-T3SS-dependent, mechanism (Krieger et al., 2014).

Krieger et al. propose a model of SIF biogenesis wherein SPI2-T3SS-secreted effectors, in particular SifA, recruit and fuse host membrane vesicles to the SCV providing components for tubule extension. PipB2 then promotes SIF extension by linking nascent SIFs to the kinesin-1 molecular motor promoting extension of SIFs outwards from the SCV along microtubules (Henry et al., 2006). Single-membrane SIFs are then converted to double-membrane SIFs by SseF and SseG (Krieger et al., 2014). The double membrane structure of SIFs would allow *Salmonella* to maintain contact with endocytosed materials (e.g., nutrients), while remaining separated from host cell cytosol (and potential host antimicrobial defenses). This model accounts for the activities of four of the eight SPI2-T3SS-secreted effectors associated with SIF biogenesis, namely SifA, PipB2, SseF, and SseG. It remains unknown how the other four effectors contribute in this model.

## SIFs link *Salmonella* to the endocytic and exocytic pathways

*S*. Typhimurium has specifically evolved to establish the SIF network, yet the role of the network is unclear. Mounting experimental evidence indicates that intracellular *Salmonella* interacts directly with the host's endocytic system. SIFs are characterized by host late endosome membrane markers LAMPs, vATPase, Rab7, LBPA, and cholesterol. Unlike late endosomes, SIF membranes are also marked by SPI2-T3SS-secreted effectors, and low concentrations of both M6PR and cathepsin D (Figure 1; Brumell et al., 2001b; Knodler and Steele-Mortimer, 2005; Mota et al., 2009; Van Engelenburg and Palmer, 2010; Schroeder et al., 2011; Young et al., 2017). Multiple reports demonstrate that SIFs likely acquire late endocytic membrane markers by sustained fusion events with the endocytic pathway (Drecktrah et al., 2008; Rajashekar et al., 2008, 2014; Krieger et al., 2014).

*Salmonella*-induced secretory carrier membrane protein 3 (SCAMP3) is also a major component of SIFs, which unlike most SIF markers is not associated with late endocytic compartments (Mota et al., 2009). SCAMP3 is primarily localized to the *trans*-Golgi network (TGN) and controls multivesicular endosome biogenesis in uninfected cells (Castle and Castle, 2005; Falguières et al., 2012). In line with this, *Salmonella* redirects exocytic transport processes and interacts with the secretory pathway (Kuhle et al., 2006; Perrett and Zhou, 2013). This interaction, in addition to the endocytic pathway (discussed below), may allow *Salmonella* to obtain nutrients for replication, collect membrane components for SCV and SIF biogenesis, or manipulate the host cell's response to infection.

It is hypothesized that SIFs allow *Salmonella* to redirect host vesicular traffic to supply intravacuolar *Salmonella* with endocytosed nutrients and membrane components to promote intravacuolar replication (Kuhle et al., 2006; Perrett and Zhou, 2013; D'Costa et al., 2015). Fluorescently labeled endosomal cargo is detected within SIF networks of infected cells (Drecktrah et al., 2008; Rajashekar et al., 2008; Zhang and Hensel, 2013) providing evidence that SIFs have access to content endocytosed by the host cell. Furthermore, it was demonstrated that the SPI2-T3SS-dependent remodeling of the host cell's endosomal transport system provides a means by which intravacuolar *S*. Typhimurium can gain access to endocytosed nutrients (Popp et al., 2015). It was recently reported that membrane components and luminal content of the SIF network are both connected to, and interchanging with, the SCV in a SPI2-T3SS-dependent manner (Krieger et al., 2014; Liss et al., 2017), allowing *S*. Typhimurium rapid access to endocytosed materials. Liss et al. (2017) also demonstrated that intravacuolar *S*. Typhimurium connected to the SIF network are significantly more metabolically active than *S*. Typhimurium in SCVs lacking connections to SIFs, suggesting that SIFs enable nutrient acquisition. Collectively, these findings suggest that *S*. Typhimurium uses the host endocytic system to expand its replicative niche, form the SIF network, and uses SIFs to gain access nutrients to promote replication within the SCV.

## Extending the SIF network

One approach to further characterize *Salmonella*'s intracellular replicative niche is to examine the host proteins associated with the SIT network. A recent study used a proteomics-based approach to enrich and characterize membranes specifically associated with the SIT network and the SCV (Vorwerk et al., 2015). The authors confirmed previous reports that *Salmonella* actively recruits host membranes for SIF formation from endosomes, lysosomes, and the trans-Golgi network (Brumell et al., 2001b; Ramsden et al., 2007; Mota et al., 2009) as they detected proteins originating from these sources in the membrane fraction enriched for the SIT network and SCV (Vorwerk et al., 2015). Proteins originating from other host compartments such as the endoplasmic reticulum (ER), nucleus, and mitochondria were also identified (Vorwerk et al., 2015).

The presence of ER proteins in association with SIFs prompts the question of if and/or how intravacuolar *Salmonella* interacts with the ER. Vorwerk et al. (2015) first reported extensive interactions between SIFs and the ER network, revealing markers commonly associated with the ER in the membrane fraction enriched for the SIT network and SCV. These included: VCP (a transitional endoplasmic reticulum ATPase), ER localized GTPases (Rab2, Rab10, Sar1A), and vesicle coat proteins associated with coatomer protein complexes I and II (COPI and COPII, respectively). Another group demonstrated that the early SCV physically interacts with the ER via ER filaments wrapped around the SCV (Santos et al., 2015). This ER-SCV interaction was observed using high-resolution ultrastructural imaging combined with immunofluorescence to give higher resolution than light or confocal microscopy alone (Narayan and Subramaniam, 2015; Santos et al., 2015). Santos et al. (2015) also showed via proteomics that COPII complexes accumulate on the early SCV membrane. COPII complexes are typically associated with transport from the ER to the Golgi in uninfected cells (Bonifacino and Glick, 2004). The accumulation of COPII complexes on the SCV membrane destabilizes the SCV membrane through an unknown mechanism, permitting *S*. Typhimurium to escape the SCV and hyper-replicate within the cytosol of epithelial cells (Santos et al., 2015). Therefore, interactions with the ER may play a critical role in determining whether *S*. Typhimurium stays within the SCV, or the SCV ruptures and *S*. Typhimurium enters the cytosol of the host cell.

Given the interactions between intravacuolar *Salmonella* and the ER (or ER derived vesicles), there is the potential that the SCV associates with the ER system via multiple contact sites (MCSs). MCSs are regions where two organelles are in close apposition (between 3 and 15 nm at ER MCSs) and permit communication between organelles (Phillips and Voeltz, 2016). The tubular ER network forms abundant MCSs with other organelles and the plasma membrane (Raiborg et al., 2015). Live-cell imaging and high-resolution ultrastructural imaging revealed membrane interactions between the ER and the early SCV resembling MCSs as early as 30 min post-invasion. This SCV-ER connection may be promoted by SCV Rab7 and ER VAP-A (Santos et al., 2015). This study reveals an unprecedented level of contact between SCVs and the ER. It is therefore possible that there are also MCSs between SIFs, ER membranes, and other host organelles, enabling intravacuolar *Salmonella* to be in contact with host cell compartments at distant intracellular locations. The evidence of proteins acquired from a range of host compartments suggests that the interactions between SIFs/SCVs and the host cell are far more expansive than previously thought.

## Conclusions

The ability to form BCVs, survive, and replicate within host cells is a common strategy among intracellular pathogens. Unique to *Salmonella*'s intracellular replicative environment is the formation of the elaborate SIT and SIF network. *S*. Typhimurium specifically develops the complex SIF network, yet their mechanisms of development and action remain unclear. There are two non-mutually exclusive hypotheses to explain SIF function: (1) SIFs collect host membrane components providing access to endocytosed compounds, including nutrients, enabling replication and membrane expansion; and (2) the SCV/SIF interconnectivity reduces exposure of intravacuolar *Salmonella* to the host defenses by diluting lysosomal enzymes inevitably acquired by the SCV. Fusion of host endocytic cargo from various intracellular locations to provide the membrane materials enabling SIF biogenesis explains both the abundance and variety of host markers found within SIF and SCV membranes.

Although recent studies have significantly advanced our understanding of *Salmonella*'s intracellular lifestyle, additional work is needed to characterize SIFs if we wish to fully understand *S*. Typhimurium's intracellular niche. Despite the large body of research regarding SIFs, several areas require further investigation. It remains unclear why *S*. Typhimurium has evolved to form complex multi-SPI2-T3SS-effector-dependent SIFs. The mechanisms underpinning SIF biogenesis still requires work to determine how all eight implicated SPI2-T3SS effectors create these unique structures. The ability to form SIFs correlates with the ability to cause disease in mouse models of infection (Stein et al., 1996), but their existence or purpose in human infections is unknown. A great deal could be learned from observing SIFs in infected tissue to address the correlation, and potential causation, between SIF biogenesis and disease phenotypes observed in mouse models of infection. It is possible to observe *Salmonella* in association with LAMPs in infected tissues (Zhang et al., 2014); the technology is already available to further investigate this matter. In conclusion, determining how SIFs form and why they are important during infection will lead to a deeper understanding of *Salmonella*'s unique replicative niche, and may reveal novel insights into other intracellular pathogens.

## Author contributions

KK drafted the manuscript; BF revised the manuscript critically for important intellectual content.

### Conflict of interest statement

The authors declare that the research was conducted in the absence of any commercial or financial relationships that could be construed as a potential conflict of interest.
